# Successful use of spesolimab in refractory pyoderma gangrenosum

**DOI:** 10.1016/j.jdcr.2026.02.026

**Published:** 2026-02-17

**Authors:** Alexa Lum, Suraj Patel, Joshua Aron, Francisca Kartono

**Affiliations:** a Michigan State University College of Osteopathic Medicine, East Lansing, Michigan; b Department of Dermatology, Corewell Health Farmington Hills Dermatology, Farmington Hills, Michigan

**Keywords:** dermatology, interleukin-36, neutrophilic dermatosis, pathergy, pyoderma gangrenosum, spesolimab

## Introduction

Pyoderma gangrenosum (PG) is a rare inflammatory neutrophilic dermatosis that typically begins as a painful erythematous papule or pustule and rapidly progresses to ulceration and necrosis, often with irregular, violaceous, undermined borders. PG is frequently associated with underlying systemic disease and autoimmune diseases such as rheumatoid arthritis and ulcerative colitis and can develop following minor trauma—a phenomenon known as pathergy.^1^ Because of this, misdiagnosis as a nonhealing ulcer can have serious consequences, as debridement may exacerbate the disease due to this pathergic response.

Approximately 85% of PG cases are of the ulcerative subtype, classically involving the lower legs. Less common subtypes include bullous, vegetative, pustular, peristomal, and superficial granulomatous forms, which may occasionally transition from 1 subtype to another.^2^ Although the exact pathogenesis remains unclear, interleukin-36 (IL-36) has been implicated as 1 of the key mediators of neutrophil chemotaxis seen with the disease. Additionally, a genetic component has been proposed, with mutations in the *PSTP1P1*/*CD2BP1* gene identified in Pyogenic Arthritis, Pyoderma Gangrenosum, and Acne and Pyoderma Gangrenosum, Acne, and Hidradenitis Suppurativa syndromes.^1^

Diagnosis can be challenging, as histopathologic findings are nonspecific and primarily serve to exclude other causes of chronic ulceration. Likewise, management of PG remains complex, as there is no established gold-standard therapy. Treatment generally involves systemic immunosuppression with medications such as cyclosporine, corticosteroids, and tumor necrosis factor alpha inhibitors, along with supportive wound care.^1^ Here, we present a case of refractory PG that demonstrated marked improvement following treatment with spesolimab after failure of multiple standard therapies.

## Case report

**Fig 1:**
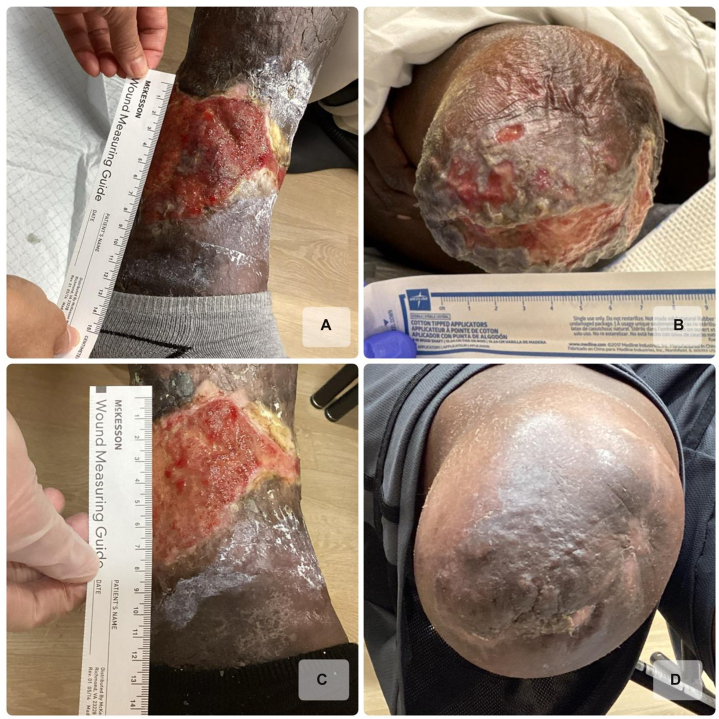
A 54-year-old female with pyoderma gangrenosum. **A** and **B,** Prior to spesolimab treatment (**[A]** Left medial lower leg, **[B]** Right lower extremity amputation site). **C** and **D,** Three months after first 600 mg infusion of spesolimab (**[C]** Left medial lower leg, **[D]** Right lower extremity amputation site).

A 54-year-old female with a past medical history of congestive heart failure, chronic kidney disease, and hypertension presented with a known diagnosis of PG on the bilateral lower extremities with onset approximately 9 years ago. In 2018, she was treated for presumed ulcers secondary to chronic venous insufficiency, without adequate response to standard therapies at that time. A punch biopsy of 1 of the left lower extremity lesions in 2020 showed a dense neutrophilic infiltrate at the wound edge, suggestive of PG. Additionally, she achieved a PARACELSUS score of 16 of 20, further indicative of a diagnosis of PG.^3^ She was initiated on doxycycline 100 mg twice daily and intralesional steroid injections without relief of symptoms. Later that year, she was started on cyclosporine 200 mg every morning and 100 mg at night, without any improvement of her ulcers. From 2021 to 2023, she tried multiple different therapies including dapsone 100 mg daily, vitamin E 800 international units daily, mycophenolate mofetil 1500 mg daily, and prednisone 60 mg daily. Despite these various therapeutic regimens, her ulcerations continued to progress, complicated by recurrent secondary infections, ultimately necessitating a right below-the-knee amputation in 2024. Following the amputation, new PG lesions were noted to develop at the surgical site. In April 2025, she presented to our office for re-evaluation and was subsequently initiated on a trial of spesolimab injections, guided by the positive results from Guénin et al^4^ and the ongoing phase 3 trial investigating spesolimab in PG.^5^ The patient received a 600 mg loading dose, followed by 300 mg subcutaneous injections administered every 4 weeks. Within 3 months of the first dose, she demonstrated remarkable improvement, including a decrease in ulcer size, reduction of her pain from a 9 of 10 to a 5 of 10, and complete closure of the ulcers at the amputation site (Fig 1).

## Discussion

IL-36 overexpression has been implicated in several neutrophilic dermatoses, including PG. Following skin injury, IL-36 cytokines are released from keratinocytes, and the innate immune system becomes activated, promoting neutrophil recruitment, activation, and degranulation.^6^ Proteases released by neutrophils further activate IL-36, leading to increased expression in proinflammatory cytokines such as interleukin-8, tumor necrosis factor alpha, interleukin-17, and interleukin-6.

Spesolimab is a humanized monoclonal immunoglobulin G1 antibody that targets and blocks the IL-36 receptor and is currently approved only for generalized pustular psoriasis. However, given our patient’s rapid improvement in PG lesion size and symptom burden, we hypothesize that spesolimab played a key role in inhibiting the IL-36–mediated inflammatory response driving her PG. Additionally, we propose that spesolimab may represent a promising therapeutic option for PG lesions, particularly in cases that are refractory to conventional immunosuppressants and/or are recurrent in nature.

Additional reports support the use of spesolimab in refractory PG, further implicating IL-36 in disease pathogenesis. Guénin et al described 2 patients who experienced substantial symptom relief and visible ulcer improvement within days of their first spesolimab infusion.^4^ Xin et al reported similar findings, with the lesions decreasing in size within 4 weeks of the first spesolimab infusion.^7^ Although the positive results demonstrated in both cases were limited by concomitant immunomodulatory treatments, spesolimab was the common variable associated with clinical improvement.

Notably, our patient achieved marked improvement with a comparatively moderate dose relative to previously published cases. In the case series by Guénin et al, patient #1 achieved significant epithelial regeneration with 4 900-mg infusions of spesolimab administered every 4 weeks over a 16-week period.^4^ In contrast, our patient demonstrated similar clinical improvement with 300-mg injections over the same interval. Further research is necessary to elucidate the role of spesolimab in PG and other IL-36–mediated disorders and to propose an optimal dosing strategy that balances efficacy and safety.

## Conflicts of interest

None disclosed.
